# Burden of cardiovascular dysfunction and outcome among term newborns having birth asphyxia

**DOI:** 10.12669/pjms.38.4.5160

**Published:** 2022

**Authors:** Waqas Shakir, Abdur-Rehman, M. Sohail Arshad, Nazia Fatima

**Affiliations:** 1 Waqas Shakir, FCPS (Pediatrics Medicine), Department of Neonatology, Children’s Hospital and The Institute of Child Health, Multan, Pakistan; 2 Abdur Rehman, FCPS Department of Neonatology, Children’s Hospital and The Institute of Child Health, Multan, Pakistan; 3 M. Sohail Arshad, FCPS Department of Pediatric Cardiology, Children’s Hospital and The Institute of Child Health, Multan, Pakistan; 4 Nazia Fatima, FCPS (Pediatric Medicine), Department of Neonatology, Children’s Hospital and The Institute of Child Health, Multan, Pakistan

**Keywords:** Cardiovascular dysfunction, Birth asphyxia, Inotropes, Echocardiography

## Abstract

**Objectives:**

To find out the burden of cardiovascular dysfunction and outcome among term newborns having birth asphyxia.

**Methods:**

This prospective observational study was conducted at The Department of Neonatology, Children’s Hospital and The Institute of Child Health, Multan from August 2020 to March 2021.A total of 171 term newborns having asphyxia were enrolled. Detailed history along with clinical and physical examination were done at the time of admission at Neonatal Intensive Care Unit (NICU). All neonates were followed up for duration of 14 days following birth. Echocardiographic patterns as well as electrocardiography grading were described among neonates with cardiovascular abnormalities.

**Results:**

Out of a total of 171 neonates, there were 94 (55.0%) male and 77 (45.0%) female. Lowe segment cesarean section was the mode of delivery in 72 (42.1%) while normal vaginal delivery was noted in 99 (57.9%). Mean gestational age was noted to be 38.3±1.8 weeks. Mean birth weight was calculated to be 2574.10±122.30 grams. Cardiovascular dysfunction was noted among 60 (35.1%) neonates as exhibited by the use of inotropes while abnormal ECHO was observed in 52 (30.4%), abnormal ECG in 27 (15.8%) and elevated CK-MB in 31 (18.1%). A total of 29 (17.0%) asphyxiated neonates died while among 60 asphyxiated neonates with cardiovascular dysfunction, 23 (38.3%) died and all remaining survived and discharged (p<0.0001).

**Conclusion:**

Cardiovascular dysfunction among asphyxiated neonates was found to be in high proportion of cases. Cardiovascular dysfunction was noted to have significant association with poor outcome.

## INTRODUCTION

Perinatal asphyxia is considered to a major cause of neonatal morbidity and mortality while late sequelae of perinatal asphyxia are a cause of major concern especially in developing countries.^1^,^2^ Perinatal asphyxia commonly affects the brain because of hypoxic-ischemic encephalopathy but other organs or systems are frequently overlooked which also bear the consequences of hypoxic-ischemic insult. Most commonly affected abnormalities of birth asphyxia involve kidneys in about 50% neonates, central nervous system 28%, cardiovascular 25% and pulmonary system in 23%.^3^ This describes that there is involvement of multi-organ dysfunction linked with perinatal asphyxia in the immediate neonatal period.

Myocardium of neonates is thought to be resistant to hypoxia^4^ but cardiac failure is one of the most important manifestations of myocardial dysfunction among cases having perinatal asphyxia.^5^ Although prevalence of severe cardiac damage is not high but relatively less severe manifestations involving heart might be frequent among neonates with asphyxia.^6^ In the past, murmur suggesting Atrioventricular insufficiency, electrocardiographic abnormality depicting myocardial ischemia, cardiogenic shock, hypotension, functional tricuspid incompetence or arrhythmia have been found to be frequent cardiovascular complications among neonates with asphyxia.^7^

In Pakistan, no study has specifically aimed at finding out cardiovascular abnormalities and outcome in birth asphyxia but data from India suggest that significant proportion of neonates (32%) were having cardiovascular dysfunction while neonates with cardiovascular dysfunction had significantly increased risk of poor outcome.^8^ This study was aimed at finding out burden of cardiovascular dysfunction and outcome in birth asphyxia. The findings of this study were thought to provide useful insights about the possible burden and types of cardiovascular involvement among neonates born with asphyxia.

## METHODS

This was a prospective observational study conducted at department of neonatology, Children’s Hospital and The Institute of Child Health, Multan from August 2020 to March 2021. Approval from Institutional Ethical Committee was taken (Ref#ERC/162, Dated: 06/04/2020). Written consent was sought from parents/guardians of all study participants.

A sample size of 171 cases was calculated considering 95% confidence interval, margin of error as 7% and incidence of cardiovascular abnormalities as 32% in birth asphyxia. A total of 171 newborns having asphyxia as Apgar score less than or equal to seven at five minutes with or without umbilical cord arterial pH below 7.2 at the time of birth or/and those who needed > 1 minute of positive pressure ventilation prior to sustained respiration or required mechanical ventilation at the time of birth were enrolled.^8^ All children were enrolled within 24 hours following birth and had gestational age between 37 to 42 weeks. Children having congenital anomalies or those who were suspected to have early-onset sepsis were not included. Children leaving against medical advice or whose parents/guardians did not give consent to be part of this study were also excluded.

Detailed history along with clinical and physical examination were done at the time of admission at Neonatal Intensive Care Unit (NICU). All neonates were followed up for duration of 14 days following birth. Gestational age, mode of delivery and indications for any intervention (if any) were recorded. Any complications before or during the labor were recorded accordingly. Post-natal history like birth asphyxia, resuscitation measures adopted at the time of birth along with Apgar score at 1^st^ and 5^th^ minute was noted. Echocardiography was done while each neonate was assessed for the existence of heart murmur, dysrhythmias, cyanosis, rise respiratory distress with systemic hypotension or signs of shock three seconds) were noted. Inotropes were initiated if neonates continued to have signs of systemic hypoperfusion even after fluid boluses up to 40 ml per kg. Transient myocardial ischemia was identified with the help of 12-lead serial ECG in the 1^st^ three days of admission. Grade-1 ECG changes were labeled as flat/inverted T-waves on one or two limb leads AVR. Grad-2 was described as flat/inverted T-waves in > 3 leads except AVR. Grad-3 was labeled as flat/inverted T-waves in > 3 leads and either ST depression or elevation more than 2-mm in at least two chest leads or more than 1 mm in at least 2 standard leads, or a Q-wave abnormality of more than 0.02 seconds or amplitude above 25% of R-wave in one anterior or three related chest leads. Grade-4 was labeled as presence or classical segmental infarction with abnormal Q-wave and markedly elevated ST segment or complete left bundle branch block. Creatinine kinase-MB (CK-MB) isoenzyme levels were noted at eight hours and 24 hours following admission while values above 92.6 U/L at 8 hours and 60 U/L at 24 hours were considered as high. Among neonates who had persistent murmurs and/or abnormal findings, ECHO was done.

SPSS version 26.0 was used for data analysis. Qualitative variables like gender, mode of delivery, use of inotropes and presence of cardiovascular involvement were represented as frequency and percentages. Echocardiographic patterns as well as electrocardiography grading were described among neonates with cardiovascular abnormalities. Poor outcome (death) was compared using chi square test among asphyxiated neonates with and without cardiovascular dysfunction. P value less than 0.05 was considered as significant. A special proforma was made to record all study information.

## RESULTS

Out of a total of 171 neonates, there were 94 (55.0%) male and 77 (45.0%) female. Lowe segment cesarean section was the mode of delivery in 72 (42.1%) while normal vaginal delivery was noted in 99 (57.9%). Mean gestational age was noted to be 38.3±1.8 weeks. Mean birth weight was calculated to be 2574.10±122.30 grams. Table-I is showing maternal and fetal characteristics of study participants.

**Table-I:** Maternal and Fetal Characteristics of Study Participants (n=171).

Characteristics		Number (%)
Gender	Male	94 (55.0%)
	Female	77 (45.0%)
Mode of Deliver	Cesarean Section	72 (42.1%)
	Vaginal Delivery	99 (57.9%)
Gestational Age	37-39	128 (74.9%)
	40-42	43 (25.1%)
Birth Weight (grams)	<2500	39 (22.8%)
	≥ 2500	132 (77.2%)

**Fig.1:**
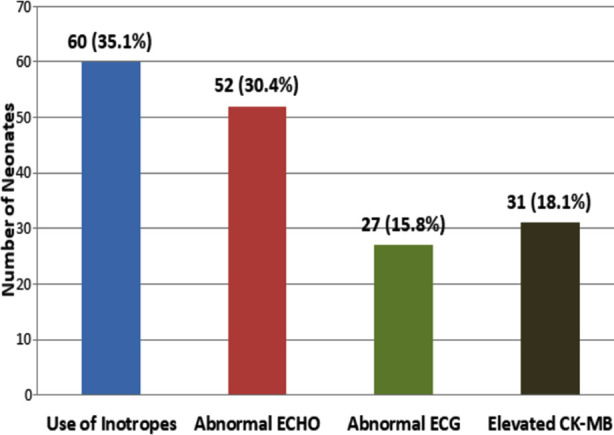
Cardiovascular Involvement among Asphyxiated Neonates (n=171).

Cardiovascular dysfunction was noted among 60 (35.1%) neonates as exhibited by the use of inotropes while abnormal ECHO was observed in 52 (30.4%), abnormal ECG in 27 (15.8%) and elevated CK-MB in 31 (18.1%). Fig.1 is showing cardiovascular system involvement noted among asphyxiated neonates.

Use of inotropes was noted to be among 60 (35.1%) neonates at day-1 of admission while it was in 41 (24.0%) at day-3, 14 (8.2%) at day-7 while none of the neonates required inotropes at day-14. Among 27 neonates with abnormal ECG, 18 were having Grade-1 ECG changes, five had Grade-2 and Grade-4 as Grade-4 changes. Persistent pulmonary hypertension was found to be the commonest echocardiographic abnormality observed in 25 (14.6%) neonates. Distribution and patterns of echocardiographic findings among asphyxiated neonates is shown in Table-II.

**Table-II:** Echocardiographic Findings among Asphyxiated Neonates.

Echocardiographic Findings	Number (%)
Persistent Pulmonary Hypertension	25 (14.6%)
Moderate Patent Ductus Arteriosus	16 (9.4%)
Moderate Patent Ductus Arteriosus + Patent Foramen Ovale	5 (2.9%)
Moderate Patent Ductus Arteriosus + Patent Foramen Ovale + Moderate Tricuspid Regurgitation	3 (1.8%)
Ventricular Septal Defect	3 (1.8%)

A total of 29 (17.0%) asphyxiated neonates died while among 60 asphyxiated neonates with cardiovascular dysfunction, 23 (38.3%) died and all remaining survived and discharged (p<0.0001).Table-III

**Table-III:** Distribution of Mortality with respect to Cardiovascular Dysfunction.

Mortality	Cardiovascular Dysfunction		P-Value

	Yes (n=60)	No (n=121)	
Yes	23 (38.3%)	6 (5.0%)	<0.0001
No	27 (61.7%)	115 (95.0%)	

## DISCUSSION

Perinatal asphyxia is considered to be a major yet preventable cause of neonatal mortality especially in developing countries.^1^ In the present study, cardiovascular dysfunction as need for the use of inotropes was noted in 35.1% asphyxiated neonates. De Dios JG et al from Spain reported cardiovascular manifestations among 19.8% of asphyxiated newborns which is lower than what we found in the present study.^9^ Shah et al found predominant majority of asphyxiated neonates (62%) who needed inotropes.^10^ Hankins and colleagues also revealed that 61% of the asphyxiated neonates needed inotropes.^11^ On the other hand Martin-Ancel et al revealed that only 4% of the asphyxiated neonates needed inotropes.^12^ All these variations could be due to different composition of study sample involving variable number of neonates with different definitions of severity levels of perinatal asphyxia.^13^ Researchers have highlighted significant presence of cardiovascular dysfunction that is secondary to neonatal asphyxia but no studies have been done to find out long-term cardiovascular outcomes.^14^ Only some researchers have highlighted persistent pulmonary hypertension to be long-term cardiovascular abnormality among asphyxiated neonates.^15^

In this study we found that abnormal ECG was found among 15.8% asphyxiated neonates whereas among these neonates, 66.7% were having Grade-1 ECG changes. Some other researchers have found a higher proportion of neonates (76.7%) with abnormal ECG findings. Rajakumar et al reveled 73.3% of the asphyxiated neonates to report ECG changes. The ECG abnormalities could be indicating myocardial ischemia secondary to birth asphyxia.^16^ Kumar PS from India recorded ECG abnormalities among 46.6% of the asphyxiated neonates.^17^

Elevated CK-MB was noted among 18.1% asphyxiated neonates. This is pretty consistent to what has previously been found by Singh et al where they revealed 14.5% of the asphyxiated neonates to have elevated CK-MB.^8^ Hankins et al reported 17% of the asphyxiated neonates to have elevation of CK-MB levels.^11^ Significance of CK-MB as a possible indicator of myocardial injury among neonates with asphyxia still needs further evaluation in the future studies.

In the present study, 30.4% asphyxiated neonates were found to have abnormal ECHO findings while most abnormality was persistent pulmonary hypertension followed by moderate patent ductus arteriosus. Linkage between meconium aspiration and persistent pulmonary hypertension has been reported in the literature^18^ but we could not report presence of meconium aspiration among present group of study cases due to limitation of our study protocol. Similarly, raised pulmonary pressure could be a contributing factor to hemodynamic instability. A study from Iran reported 62% of the asphyxiated neonates to have patent ductus arteriosus.^19^ Some studies have indicated patent arterial duct to be a factor responsible for causing cardiovascular dysfunction and persistent pulmonary hypertension.^20^,^21^

In the present research, we found that among 60 asphyxiated neonates with cardiovascular dysfunction, 23 (38.3%) died. Singh et al reported mortality among 42% of neonates with cardiovascular dysfunction while Shah et al found 64% of asphyxiated neonates with cardiovascular abnormalities to report adverse outcomes or death.^8^ Among asphyxiated neonates, high index of suspicion should be made for the early detection of cardiovascular abnormalities as timely identification and management can lead to improved outcome.Being the 1^st^ study from Pakistan that looked into cardiovascular dysfunctions among asphyxiated neonates is one of the major strength of this study.

### Limitations of the study:

As this was a single study with no comparator or control groups, further multicenter studies with comparative designs should be conducted. We did not plan to monitor the surviving neonates for possible long-term outcomes. More studies are required to find out correlation of different stages of asphyxia with different cardiovascular manifestations among asphyxiated neonates.

## CONCLUSION

Cardiovascular dysfunction among asphyxiated neonates was found to be in high proportion of cases. Cardiovascular dysfunction was noted to have significant association with poor outcome. Early screening as well as identification of cardiovascular system among asphyxiated neonates might help decreasing the burden of morbidity and mortality related to cardiovascular dysfunction among asphyxiated neonates.

### Authors’ Contribution:

**WS:** Data Collection, Drafting, Responsible for Data’s Integrity

**AR:** Methodology, Supervision, Proof Reading

**MSA:** Review of Literature, Data Analysis

**NF:** Data Interpretation, References.
